# Wesen, Ursache und Behandlung der Zuckerkrankheit (Diabetes Mellitus)

**Published:** 1899-03

**Authors:** 


**Wesen, Ursache und Behandlung der Zuckerkrankheit (Diabetes Mellitus).** Von Dr. ALBERT LENNÉ. Pp. iv., 152. Berlin: S. Karger. 1898.

Dr. Lenné has put together a clear and readable account of diabetes for physicians and students. As the outcome of a large practical experience, he finds little success in the use of drugs, though he considers that opium is of some value, and long-continued courses of alkalies when commenced early enough may indirectly produce good results. There does not appear any undue praise of the virtues to be found in the thermal springs of Neuenahr, where the writer practises, though the benefits of a course at a pleasant watering-place are not ignored. Of the value of rest for mind and body and the care of the general health he speaks strongly. As to other treatment, Dr. Lenné seems to rely chiefly on a diet carefully regulated from time to time according to the assimilative powers of his patients, and he prefers whole bread, in quantities such as can be easily dealt with in the body, to any of the artificial substitutes.

A review of modern theories of the pathology of diabetes is given in a succinct form, and extremely full tables of the sugar and starch contents of all possible articles of diet are added.

On the whole it is an interesting and well-written book, and in many points well up to date. Moreover, it possesses the luxury of a capital index, which adds considerably to its value.

